# Expanding the PURA syndrome phenotype: A child with the recurrent *PURA* p.(Phe233del) pathogenic variant showing similarities with cutis laxa

**DOI:** 10.1002/mgg3.1562

**Published:** 2020-12-04

**Authors:** Valeria Cinquina, Claudia Ciaccio, Marina Venturini, Riccardo Masson, Marco Ritelli, Marina Colombi

**Affiliations:** ^1^ Division of Biology and Genetics Department of Molecular and Translational Medicine University of Brescia Brescia Italy; ^2^ Developmental Neurology Unit Fondazione IRCCS Istituto Neurologico Carlo Besta Milan Italy; ^3^ Division of Dermatology Department of Clinical and Experimental Sciences Spedali Civili University Hospital Brescia Brescia Italy

**Keywords:** cutis laxa, hypotonia, neurodevelopmental delay, *PURA*, PURA syndrome, whole exome sequencing

## Abstract

**Background:**

PURA syndrome is rare autosomal dominant condition characterized by moderate to severe neurodevelopmental delay with absence of speech in nearly all patients and lack of independent ambulation in many. Early‐onset problems include excessive hiccups, hypotonia, hypersomnolence, hypothermia, feeding difficulties, recurrent apneas, epileptic seizures, and abnormal nonepileptic movements. Other less common manifestations comprise congenital heart defects, urogenital malformations, and various skeletal, ophthalmological, gastrointestinal, and endocrine anomalies. Up to now, 78 individuals with PURA syndrome and 64 different pathogenic variants have been reported, but no clear‐cut genotype‐phenotype correlations have emerged so far. Herein, we report the clinical and molecular characterization of a 3‐year‐old girl with severe hypotonia, global developmental delay, and soft, loose skin, who came to our attention with a suspicion of cutis laxa (CL), which denotes another condition with variable neurodevelopmental problems.

**Methods:**

Amplicon‐based whole exome sequencing was performed, and an in‐house pipeline was used to conduct filtering and prioritization of variants. New prediction algorithms for indels were used to validate the pathogenicity of the *PURA* variant, and results were confirmed with the Sanger method. Finally, we collected clinical and mutational data of all PURA syndrome patients reported yet and compared the clinical features with those of our patient.

**Results:**

Clinical evaluation and biochemical investigations excluded CL and prompted to perform whole exome sequencing, which confirmed the absence of pathogenic variants in all CL‐related genes and revealed the known *PURA* c.697_699del, p.(Phe233del) variant, identified hitherto in seven additional children with PURA syndrome.

**Conclusions:**

Our data expand the phenotypic spectrum of PURA syndrome by showing that it can be regarded as a differential diagnosis for cutis laxa in early infancy. Our patient and literature review emphasize that a wide clinical variability exists not only between individuals with different *PURA* variants, but also among patients with the same causal mutation.

## Graphical abstract

PURA syndrome is a developmental encephalopathy presenting with neonatal hypotonia, feeding difficulties, global developmental delay, intellectual disability, and frequent apnea and epilepsy. We describe a 3‐year‐old girl with severe hypotonia, neurodevelopmental delay and soft, loose skin, in whom whole exome sequencing disclosed a known pathogenic *PURA* variant. Our data expand the phenotypic spectrum of PURA syndrome by showing that it can be regarded as a differential diagnosis for cutis laxa in early infancy.

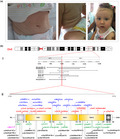

## 1 INTRODUCTION

Purine‐rich element‐binding protein A (PURA)‐related disorders (MIM #616158) are rare conditions estimated to account for less than 1% of cases of neurodevelopmental delay. They are variably characterized by moderate to severe intellectual disability (ID) and early‐onset issues including hypotonia, motor delay, feeding difficulties, hypersomnolence, recurrent central and obstructive apneas, speech delay, and abnormal nonepileptic movements. Many individuals have epilepsy and in some cases seizures may be intractable. Other less common manifestations comprise congenital heart defects, skeletal, urogenital, ophthalmological, gastrointestinal, and endocrine anomalies; mild cutaneous involvement as well as some craniofacial features were also reported (Reijnders et al., 2017).

PURA‐related disorders are caused by genetic alterations involving *PURA* (MIM *600473), which is a single‐exon gene that encodes Pur‐α, a highly conserved, ubiquitously expressed multifunctional protein consisting in an N‐terminal glycine‐rich domain (6–57 aa), three central conserved sequence‐specific repeats, Pur repeat I‐II‐III (60–279 aa), and a C‐terminal Gln/Glu‐rich domain (282–303 aa) (Graebsch et al., 2009; Weber et al., 2016). Pur‐α essential for normal postnatal brain development as it is involved in neuronal proliferation, dendrite maturation, mRNA transport to translation sites in hippocampal neurons, and formation and maturation of myelin (Hokkanen et al., 2012; Johnson et al., 2006, 2013; Khalili et al., 2003; White et al., 2009). Individuals with ID and *PURA* haploinsufficiency were first identified within the 5q31.3 deletion syndrome (Bonaglia et al., 2015; Brown et al., 2013; Hosoki et al., 2012; Shimojima et al., 2011, 2018). Later, whole exome sequencing (WES) allowed the identification of de novo pathogenic sequence variations in the gene (Hunt et al., 2014; Jezela‐Stanek et al., 2020; Lalani et al., 2014; Lee et al., 2018; Mayorga et al., 2018; Okamoto et al., 2017; Qiao et al., 2019; Reijnders et al., 2018; Rezkalla et al., 2017; Rodríguez‐García et al., 2020; Tanaka et al., 2015; Trau & Pizoli, 2020). This latter condition due to point mutations is currently termed PURA syndrome (Reijnders et al., 2017).

Cutis laxa (CL) is another group of disorders with variable neurodevelopmental problems, especially the *ATP6V0A2*‐ and the *PYCR1*‐related CL subtypes (Dimopoulou et al., 2013; Reversade et al., 2009; Van Maldergem et al., 2015). *ATP6V0A2*‐related CL, a.k.a. autosomal recessive CL type 2A (ARCL2A, MIM #219200), spans a phenotypic spectrum including the Debré‐type at the severe end and wrinkly skin syndrome (WSS, MIM #278250) at the mild end. Children with the Debré‐type have more severe developmental and neurologic abnormalities and a less severe cutaneous phenotype than children with WSS. At birth, hypotonia, over‐folded skin, congenital hip dislocation, inguinal hernias, enlarged fontanelles, and distinctive facial features (droopy skin on cheeks, marked nasolabial folds, prominent large nasal root, down‐slanted palpebral fissures) are usually present. Nearly all affected children show delayed developmental milestones and ID often associated with cortical and cerebellar malformations ranging from mild cerebellar vermis hypoplasia to classic Dandy‐Walker malformation, including severe hypoplasia and upward rotation of the vermis, cystic enlargement of the fourth ventricle, and enlarged posterior fossa. Many patients have a degenerative course with cognitive decline, as well as neurologic regression (with or without seizures) including spasticity and cerebellar signs and symptoms (ataxia and slurred speech). WSS embraces many features of the Debré‐type CL, but usually children have a milder developmental delay without subsequent neurodegeneration. Diagnosis of ARCL2A is based on the characteristic skin findings, serum sialotransferrin isoelectric focusing (IEF), and genetic testing of *ATP6V0A2* (MIM *611716) (Van Maldergem et al., 2015). Pathogenic *PYCR1* (MIM *179035) variants cause autosomal recessive CL type 2B and 3B (ARCL2B, MIM #612940 and ARCL3B, MIM #614438). Although patients with ARCL2B show a phenotype that shares many similarities with ARCL2A, they often have a more progeroid appearance with a common facial gestalt (triangular face, large everted ears, and hypomimia) and hypoplasia of the corpus callosum. About 95% of affected individuals have ID. Patients who additionally have corneal clouding or cataracts are considered to have ARCL3B (Dimopoulou et al., 2013; Reversade et al., 2009).

Herein, we report on a 3‐year‐old child with severe hypotonia and global developmental delay with a cutaneous phenotype suggestive of CL, in whom WES disclosed a known pathogenic *PURA* variant, and review all patients with PURA syndrome described so far.

## 2 PATIENTS AND METHODS

### 2.1 Ethical compliance

The patient was evaluated both at the Developmental Neurology Unit, IRCCS Foundation, “Carlo Besta” Neurological Institute of Milan, and at the specialized outpatient clinic for the diagnosis of hereditary connective tissue disorders, that is, the Ehlers–Danlos syndrome and Inherited Connective Tissue Disorders Clinic (CESED), at the University Hospital Spedali Civili of Brescia. This study followed the Declaration of Helsinki's principles and was approved by the local ethics committees. Genetic testing was achieved in compliance with the Italian legislation on diagnostic tests and the patient's parents provided written informed consent for the publication of clinical data and photographs. Whole exome sequencing was performed at the Division of Biology and Genetics, Department of Molecular and Translational Medicine, of the University of Brescia.

### 2.2 Molecular analyses

Genomic DNA from the proband was extracted from peripheral blood leukocytes by standard procedures. To identify the molecular defect in the proband, we performed WES by using the Ion Proton platform and the AmpliSeq technology following the manufacturer's recommendations (Thermo Fisher Scientific). Briefly, libraries template preparation was achieved using the Ion PI Hi‐Q OT2 200 kit on the Ion OneTouch 2 and sequencing run was performed with the Ion PI Hi‐Q Sequencing 200 kit. The templated Ion Sphere Particles (ISP) were enriched for positive ISP using the Ion OneTouch ES and sequenced on Ion Proton with the Ion PI chip v3. Basecalling and sequence alignment against hg19 genome assembly were performed using Ion Torrent Suite software 5.12, and genetic variants were identified using Torrent Suite Variant Caller pipeline 5.12. Variants were annotated with ANNOVAR (Wang et al., 2010) and filtered with a standardized in‐house pipeline, as previously described (Ritelli et al., 2019). To evaluate the putative pathogenicity of the *PURA* deletion variant (NM_005859.4), we used the following mutation prediction tools for indels: SIFT (Sim et al., 2012), PROVEAN (Choi et al., 2012), VEST (Douville et al., 2016), and MutPred2 (Pagel et al., 2019).

The de novo occurrence of the pathogenic *PURA* variant identified by WES was confirmed by Sanger sequencing with a specific primer set. The sequences were analyzed with Sequencer 5.1 software (Gene Codes Corporation) and variants annotated according to the Human Genome Variation Society (HGVS) nomenclature by using the Alamut Visual software version 2.16 (www.interactive‐biosoftware.com).

## 3 RESULTS

### 3.1 Clinical data

**FIGURE 1:**
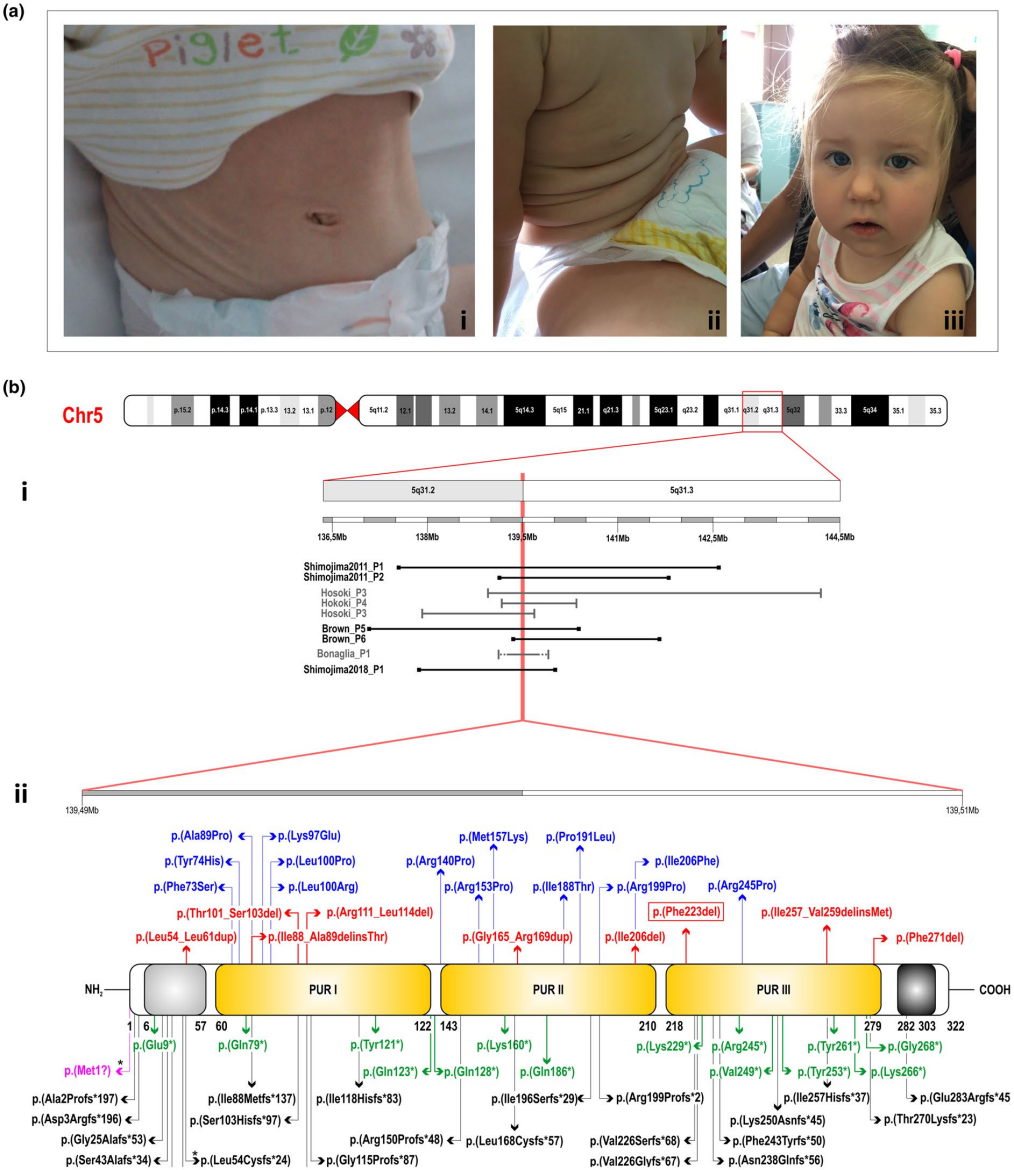
Clinical findings of the patient and mutational spectrum involving *PURA*. (A) Redundant skin folds, more prominent at thoracic and abdominal areas (i, ii) and dysmorphic features: mild myopathic face, high anterior hairline, almond‐shaped palpebral fissures, and prominent well‐defined philtrum (iii). (B) The graphic shows a schematic representation of the human chromosome 5 that contains *PURA* on locus 5q31.3 (Grch37/hg19:139,493,694–139,505,204; NM_005859.5; NP_005850.1). (i) Horizontal bars represent the different chromosomal deletions involving the 5q31.2‐q31.3 region previously reported in literature (black and grey). The dashed bars specify the approximately breakpoints of the deletions. The vertical red line represents the smallest region of overlap containing *PURA*. (ii) PURA structure and protein domains: in light grey the N‐terminal glycine‐rich domain (6–57 aa), in yellow three central conserved sequence‐specific repeats, Pur repeat I‐II‐III, (60–279 aa), and in black a C‐terminal Gln/Glu‐rich domain (282–303 aa). The different *PURA* pathogenic missense variants (in blue) and small deletions/insertions (in red) described thus far in literature are shown over the diagram. Nonsense (in green), start‐loss (in pink), and frameshift (in black) variants are shown under the diagram. *Represent two distinct variants that were both predicted to give rise to the same protein product. The variant identified in the present patients is boxed

We report the case of a girl, first child of healthy non‐consanguineous parents. She was born at 41 w + 3 after an unremarkable pregnancy; birth parameters were within the normal range (length 52.5 cm, weight 3300 g, head circumference 35 cm) and she reported no perinatal distress (APGAR 9/10). Poor sucking and deglutition impairment were noted in the first days of life, associated with desaturation and several bradycardia crises; a nasogastric tube was, therefore, inserted and used to feed the baby for the first 2 months. In that period, a cranial ultrasound (US), heart US, laryngo‐tracheo fibroscopy, and auditory brainstem response were performed, giving normal results. Also, a first genetic screening (conventional karyotype, array‐CGH, and chromosome 15 methylation test for Prader–Willi syndrome) was normal. She was first referred to our center at the age of 13 months for hypotonia and global developmental delay. Growth was normal (L −1.1 SD, W −0.8 SD, and HC −0.6 SD); she showed redundant skin folds, more prominent at thoracic, abdominal, and inferior limbs areas suggestive of CL (Figure 1A). Neurodevelopmental stages appeared severely delayed: language was absent and motor acquisitions were extremely poor, as the girl was still not able to sit without support. At clinical examination, she presented marked hypotonia, hypermobility of small joints (Beighton score BS 4/9), and drooling; marked startle response to tactile or auditory stimulation was also noted. Dysmorphic features included mild myopathic face with hypomimia, high anterior hairline, almond‐shaped palpebral fissures, and a prominent well‐defined philtrum (Figure 1A). Additional tests were performed to investigate the case. Brain magnetic resonance imaging revealed only mild myelination delay and electroneurography/electromyography and electroencephalogram were normal. A wide metabolic screening, which included plasma amino acids, acylcarnitines, copper, ceruloplasmin, vitamin D, urinary organic acids, and mucopolysaccharides, gave normal results. Given the peculiar skin presentation (Figure 1A), in order to investigate congenital disorders of glycosylation with cutis laxa, sialotransferrin IEF was performed, which showed normal transferrin distribution. Skin biopsy with orcein staining was, therefore, not performed. Global development improved very slowly over time until the age of 3 years, when the girl was able to sit alone and social smile was observed; marked hypotonia as well as skin wrinkles were still present and no expressive language evolution was noted, while she appeared able to understand extremely simple orders. Despite the peculiar skin presentation, in view of patient's clinical features and instrumental and biochemical analyses, we excluded with great confidence a diagnosis of CL. In particular, the most plausible candidates, namely the *ATP6V0A2*‐ and the *PYCR1*‐related subtypes, as well as the other forms of CL, were ruled out for the absence of more than a few distinguishing hallmarks, including their distinctive facial gestalt. On that account, we performed amplicon‐based WES to reach a molecular diagnosis among the numerous neurodevelopmental disorders with ID.

### 3.2 Molecular findings

Summary results of WES are reported in Table S1. After application of a standardized in‐house pipeline (Ritelli et al., 2019), and considering only variants previously associated with a disease phenotype, five candidate genes were identified (Table S2). Among these, WES did not disclose any pathogenic variant in all CL‐related genes, which resulted fully covered as they were also analyzed separately to confirm the absence of any variant of unknown significance, but revealed the known NM_005859.4 (*PURA*):c.697_699del variant (rs786204835), which causes the deletion of a highly conserved amino acid residue [NP_005850.1(PURA):p.(Phe233del)] within the PUR‐III domain of the protein (Figure 1B). SIFT (Sim et al., 2012), PROVEAN (Choi et al., 2012), VEST (Douville et al., 2016), and MutPred2 (Pagel et al., 2019) prediction algorithms for indels all agreed to define this variant as deleterious. Furthermore, by using the Varsome tool (Kopanos et al., 2019), which allows the interpretation of variants according to the American College of Medical Genetics and Genomics (ACMG) guidelines (Richards et al., 2008), the p.(Phe233del) *PURA* variant was classified as pathogenic (ACMG class 5). Segregation study by Sanger sequencing confirmed the presence of the variant and verified that it arose de novo (Figure 1), thus, allowing for a diagnosis of PURA syndrome.

## 4 DISCUSSION

Due to extensive clinical and genetic heterogeneity of ID syndromes, the diagnostic process might be very challenging, even for expert clinicians. During the last years, the clinical model of ID syndrome recognition has entirely changed. Prior to the introduction of next generation sequencing, molecular tests were ordered based on clinicians’ suspicion from clinical examination and patients’ history (“phenotype‐first” approach); nowadays a “genotype‐first” model of genetic assessment is often favored. Still, detailed phenotypical evaluation of patients is critical for the process of diagnosis. In this report, we described the clinical features and the molecular diagnostic resolution of a child referred to our clinic with a suspicion of CL. Detailed clinical evaluation and biochemical investigations excluded CL and prompted to perform WES, which revealed a de novo pathogenic *PURA* variant, hence, concluding the diagnostic process.

Up to now, 79 patients (including ours) with PURA syndrome and 64 different point mutations, that is, missense (14), nonsense (14), small deletions/insertions (9), start‐loss (2), and frameshift (25), have been reported (Hunt et al., 2014; Jezela‐Stanek et al., 2020; Lalani et al., 2014; Lee et al., 2018; Mayorga et al., 2018; Okamoto et al., 2017; Qiao et al., 2019; Reijnders et al., 2018; Rezkalla et al., 2017; Rodríguez‐García et al., 2020; Tanaka et al., 2015; Trau & Pizoli, 2020) together with nine patients with the 5q31.3 deletion syndrome (Bonaglia et al., 2015; Brown et al., 2013; Hosoki et al., 2012; Shimojima et al., 2011, 2018) (Figure 1B and Table S3).

**FIGURE 2:**
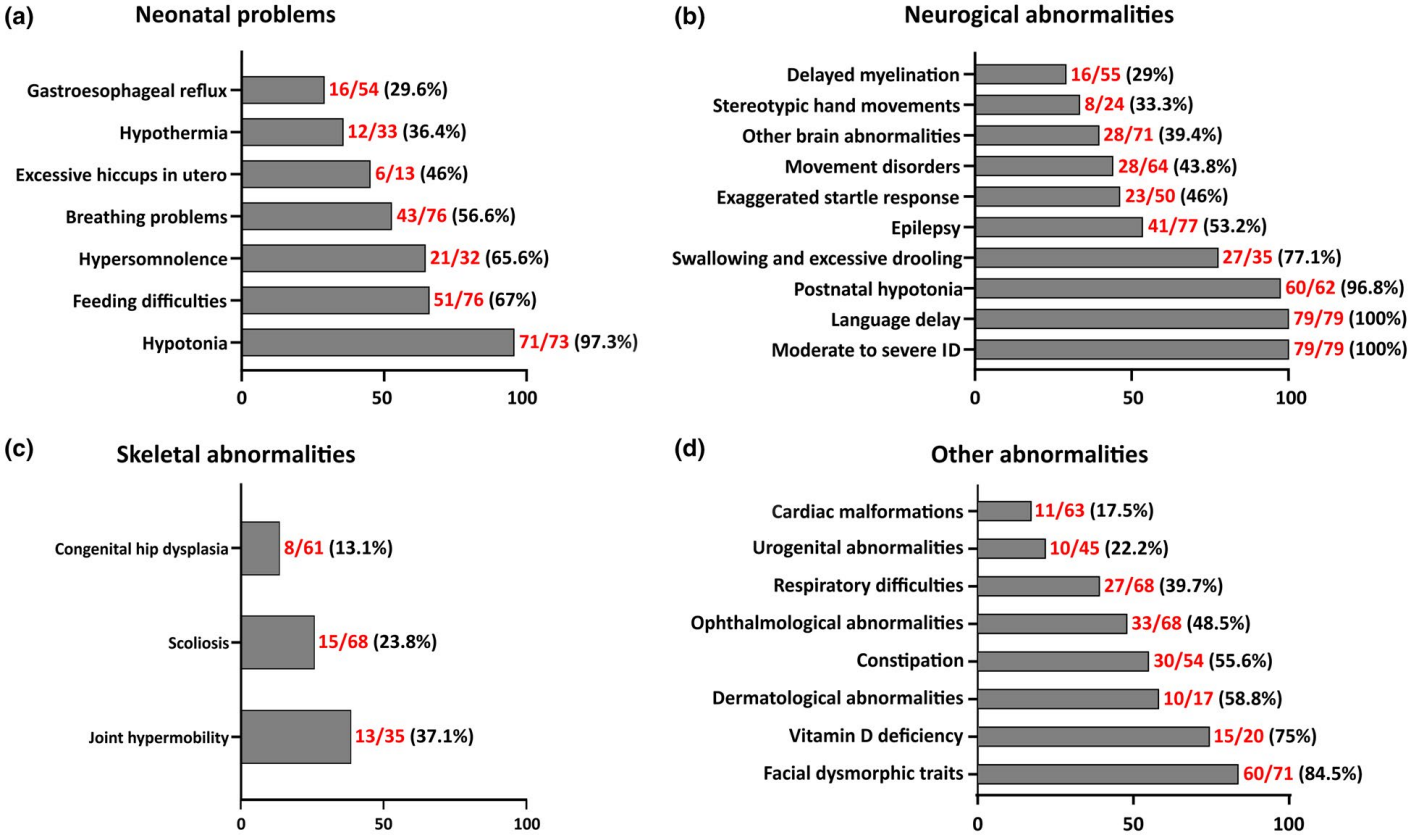
Frequencies of clinical features of all PURA syndrome patients reported so far. (a) Neonatal problems. (b) Neurological abnormalities. Other brain anomalies include white matter abnormalities, prominent periventricular spaces, parenchymal atrophy, widening of lateral ventricles, and underdeveloped rostrum of the corpus callosum. (c) Skeletal abnormalities. (d) Other abnormalities. Cardiac malformations include ventricular septal defects, aberrant left subclavian artery, pulmonary stenosis, and ductus arteriosus. Urogenital abnormalities include cryptorchidism, kidney stones, congenital hydronephrosis with megaureter, and urinary reflux. Dermatological abnormalities include soft skin and cutis laxa. Facial dysmorphic traits include myopathic face, full cheeks, high anterior hairline, abnormal shape of the eye (shorter palpebral fissures, eversion of lower lateral eyelids), and prominent, well‐defined philtrum. The ratio indicates the number of patients presenting the specific feature out of the total number of patients in whom the feature was investigated

Among the different *PURA* sequence variations, nine variants were recurrent, among which the p.(Phe233del) recognized in our child that was identified in seven additional patients (Hunt et al., 2014; Lee et al., 2018; Reijnders et al., 2018; Tanaka et al., 2015) (Table 1 and Table S3). Different mechanisms based on the various types of mutations, that is, a dominant negative effect for structural variants and functional haploinsufficiency for truncating mutations, have been suggested (Hunt et al., 2014; Lalani et al., 2014); however, no clear‐cut genotype‐phenotype correlations have been recognized yet. Reijnders et al. (2018) who classified variants in different subtypes, based on a homology model derived from the crystal structure of the drosophila Pur‐α homolog, and compared the patients’ phenotypes subdivided by mutation class, verified that both the phenotypic variability and severity were not related neither to the type nor to the localization of variants, suggesting the involvement of other genetic and biological mechanisms. The absence of reliable genotype–phenotype correlations and the existence of a highly variable, wide‐ranging clinical spectrum are corroborated also by the present patient's clinical features that we compared with those of the other individuals with the p.(Phe233del) variant and with all PURA syndrome patients reported so far (Table 1, Figure 2).

**Table 1:** Clinical features of individuals with the c.697_699del, p.(Phe233del) *PURA* variant

Citation	Present patient	Hunt et al. (2014)	Tanaka et al. (2015)	Reijnders et al. (2018)			Lee et al. (2018)	
		P4	P4	P4	P5	P14	P4	P6
**Neonatal problems**								
Excessive hiccups in utero	−	−	NA	−	+	NA	NA	NA
Hypotonia	+	+	+	+	+	+	+	+
Feeding difficulties	+	+	NA	+	+	−	+	+
Gastroesophageal reflux	+	−	NA	−	−	NA	+	+
Breathing problems	+	+	NA	+	+	−	−	+
Hypersomnolence	−	NA	NA	+	+	NA	NA	NA
Hypothermia	−	+	NA	NA	+	NA	NA	NA
**Neurological abnormalities**								
Moderate to severe ID	+	+	+	+	+	+	+	+
Language delay	+	+	NA	+	+	+	NA	NA
Postnatal hypotonia	+	+	+	+	+	+	+	+
Swallowing and excessive drooling	+	NA	NA	+	+	+	NA	NA
Movement disorders	−	+	NA	−	+	−	+	+
Stereotypic hand movements	−	NA	NA	+	+	NA	NA	NA
Exaggerated startle response	+	NA	NA	−	+	+	−	−
Epilepsy	−	+	−	−	+	+	−	+
Delayed myelination	+	+	−	+	+	+	−	NA
Other brain abnormalities	−	+	+	−	−	−	+	NA
**Skeletal abnormalities**								
Scoliosis	−	NA	NA	−	+	−	−	−
Congenital hip dysplasia	−	NA	NA	−	+	−	−	−
Joint hypermobility (BS)	+ (4/9)	NA	NA	−	+ (NA)	NA	−	−
**Facial dysmorphisms**								
Myopathic face	+	+	+	+	+	−	−	NA
Full cheeks	+	+	+	+	+	+	−	NA
High anterior hairline	+	+	+	+	−	+	−	NA
Abnormal shape of the eyes	+	+	+	+	−	+	+	NA
Prominent, well‐defined philtrum	+	+	+	−	−	−	+	NA
**Other**								
Constipation	−	NA	NA	+	+	−	−	+
Respiratory difficulties	−	+	NA	+	−	−	−	+
Cardiac abnormalities	−	NA	NA	−	−	+	−	+
Urogenital abnormalities	−	NA	NA	−	−	−	NA	NA
Ophthalmological abnormalities	−	−	+	+	+	+	+	−
Dermatological abnormalities	+	NA	NA	−	+	−	NA	NA
Vitamin D deficiency	−	+	NA	+	+	+	NA	NA

Abbreviations: BS, Beighton Score; ID, intellectual disability; N, number of patients presenting the investigated feature; NA, not available; T, total number of patients in whom the feature was investigated.

Concerning the p.(Phe233del) deletion, the first patient with this specific mutation (P4 in Table 1), reported by Hunt et al. (2014), presented severe ID with absent speech, hypotonia, feeding difficulties, breathing problems, hypothermia, exaggerated startle response, early onset epilepsy, delayed myelination and cerebral atrophy, low vitamin D levels, and was still not able to walk autonomously at 6.5 years. The child (P4) described by Tanaka et al. (2015) also showed severe hypotonia together with cortical visual impairment and periventricular leukomalacia. Next, Reijnders et al. (2018) demarcated more than a few novel findings in three patients with this variant, that is, ophthalmological abnormalities and drooling (present in all); hypersomnolence, constipation, and movement complaints (P4 and P5), excessive hiccups in utero, scoliosis, hip dysplasia, pes planus, joint hypermobility, low bone mineralization, and soft skin (P5), and ventricular septal defects (P14). Additionally, gastroesophageal reflux was reported in two patients (P4 and P6) by Lee et al. (2018). Compared to the earlier reported patients, shared clinical features in our child comprised hypotonia, neonatal feeding and breathing difficulties, gastroesophageal reflux, ID with absence of speech, exaggerated started response, delayed myelination, drooling, and joint hypermobility, and facial dysmorphisms (Table 1), whereas hypersomnolence, hypothermia, movement disorders, epileptic seizures as well as cardiac, skeletal, urogenital, ophthalmological, and endocrine anomalies were all not present. The most distinctive (and also confusing) issue was the patient's skin, which was soft, inelastic with marked, redundant skin folds. Of note, the first description of a cutaneous involvement in PURA syndrome was the large cohort published by Reijnders et al. (2018), in which soft skin was noticed in 46% of patients. Very recently, Jezela‐Stanek et al. (2020) published a 4‐year‐old female with a *PURA* start‐loss variant and hyperextensible and loose skin. Hence, it seems reasonable that cutaneous anomalies (including cutis laxa) in PURA syndrome might be more frequent than reported hitherto, but further reports are expected to confirm this hypothesis.

Overall, the present patient and detailed review of phenotypic data reported yet (Figure 2) further delineate the clinical spectrum of PURA syndrome and should assist early recognition and differential diagnosis on a “phenotype‐first” approach,” which was reported as difficult in daily clinical practice. We confirm that the clinical hallmarks of the disorder are moderate to severe ID (100%) with verbal language delay (100%) and neonatal hypotonia (97.3%), which usually associates with feeding difficulties (67%), gastroesophageal reflux (29.6%), breathing problems (56.5%), hypersomnolence (65.6%), and less frequently with hypothermia (36.4%). Most individuals remain nonverbal, but many have better receptive language than expressive language and can follow simple instructions (Reijnders et al., 2018). Hypotonia after the neonatal period (96.8%), swallowing and excessive drooling (77.1%), constipation (55.6%), early‐onset epilepsy (53.2%), ophthalmological anomalies (48.5%) (strabismus, refractive errors), stereotypic hand movements (33.3%), and other movement disorders (43.8%) are also common traits. Respiratory difficulties (39.7%), delayed myelination (29%) and other brain anomalies (39.4%) [white matter abnormalities, prominent periventricular spaces, parenchymal atrophy, widening of lateral ventricles, underdeveloped rostrum of the corpus callosum (Reijnders et al., 2018)], skeletal anomalies such as joint hypermobility (37.1%), scoliosis (23.8%), and congenital hip dysplasia (13.1%), urogenital anomalies (22.2%) [cryptorchidism, kidney stones, congenital hydronephrosis with megaureter, urinary reflux (Reijnders et al., 2018)], and cardiac malformations (17.5%) [ventricular septal defects, aberrant left subclavian artery, pulmonary stenosis, ductus arteriosus (Reijnders et al., 2018)] are instead less frequent. Endocrine abnormalities, mostly low vitamin D levels (75%) and less commonly aberrant sex and thyroid hormone levels and abnormal cortisol response, are also reported (Reijnders et al., 2018). It should be noted that some of these clinical features, including cutaneous issues (58.8%), likely have a different incidence than observed, since not all individuals have been investigated for these anomalies (Figure 2).

Regarding facial dysmorphisms, no prominent similarities have been observed among the first published patients, but recently Reijnders and colleagues performed a computational analysis of available photographs, either presented in their work or previously published, and compared it to the average image based on healthy individuals (Reijnders et al., 2018). This analysis showed that myopathic face, full cheeks, and high anterior hairline, represent recurrent features, possibly denoting *PURA* syndrome. Besides, slightly abnormal shape of the eyes (e.g., shorter palpebral fissures, eversion of lower lateral eyelids) and prominent, well‐defined philtrum were also present in a relatively broad number of individuals. Globally, at least one of these facial dysmorphic traits was recognized in 84,5% of patients (Figure 2). The facial gestalt of our child, who showed mild myopathic face with typically open mouth appearance, high anterior hairline, almond‐shaped palpebral fissures, and prominent, well‐defined philtrum, corroborates the computational modeled PURA face, which might be of help for clinicians for a better recognition of this disorder, also including the possible differential diagnosis with CL, as it happened in our patient.

## 5 CONCLUSION

In conclusion, our findings expand the phenotypic spectrum of PURA syndrome by underlining, for the first time, that it can be regarded as a differential diagnosis for CL in early infancy. Furthermore, given the extensive clinical and genetic heterogeneity of multiple ID syndromes, the presented findings highlight the importance of a “genotype‐first” approach in the genetic era of WES for a correct diagnosis of neurodevelopmental disorders.

## CONFLICT OF INTEREST

All authors declare that there is no conflict of interest concerning this work.

## AUTHORS’ CONTRIBUTIONS

MC and MR conceived the study and edited and coordinated the manuscript. MV, MC, CC, and RM recruited and made the clinical diagnoses of the patient and performed genetic counseling and follow‐up; the manuscript was drafted by VC and MR, who provided the experimental analysis, organized data contents, researched the literature, and prepared the manuscript; VC, MR, CC, RM completed the figures and tables. All authors discussed, read, and approved the manuscript.

## Supporting information

Table S1‐S3‐Fig S1
