# Supplementary material for: Expanding the PURA syndrome phenotype: A child with the recurrent PURA p.(Phe233del) pathogenic variant showing similarities with cutis laxa
Source: Mol Genet Genomic Med. 2020 Dec 4;9(1):e1562. doi: 10.1002/mgg3.1562 (PMC7963414; doi:10.1002/mgg3.1562)
Supplement: Supplementary file 1 — Table S1‐S3‐Fig S1 [file MGG3-9-e1562-s001.doc]

**Supplementary Materials**

| **Supplementary Table 1.** Total variants after filtering of whole exome sequencing data | | | |
| --- | --- | --- | --- |
| **Pipeline** | **Total (Het/Hom)** | **SNVs (Het/Hom)** | **INDELs (Het/Hom)** |
| **Rare (MAF ≤-0.01)** quality | 1450 (1316/134) | 1343 (1258/85) | 107 (58/49) |
| **Functional variants**  (missense, stop- or splice-affecting, and CDS indels) | 587 (518/69) | 480 (460/20) | 107 (58/49) |
| **Not in internal WES database** | 429 (380/49) | 340 (332/8) | 89 (48/41) |
| **Deleterious:**  DANN > 0.95, M-CAP>0.025, and CADD > 20 | 234 (185/49) | 145 (137/8) | 89 (48/41) |
| **Best candidates in genes with**  RVIS < -0.24 and GDI < 13.84 | 95 (81/14) | 69 (66/3) | 26 (15/11) |

| **Supplementary Table 2.** Best candidate genes after selecting variants by ClinVar Phenotypes | | | | | | | |
| --- | --- | --- | --- | --- | --- | --- | --- |
| **Gene** | **Variant(s)** | | **Effect(s)** | **dbSNP** | **Inheritance model** | **Genotype** | **Clinvar Phenotype** |
| *PURA* | c.697_699del | p.(Phe233del) | Deletion | rs786204835 | AD | Het | Mental retardation |
| *BBS9* | c.1280C>T | p.(Ala427Val) | Missense | rs138072724 | AR | Het | Bardet-Biedl syndrome 9 |
| *BMPER* | c.1663C>T | p.(Arg555Trp) | Missense | rs10249320 | AR | Het | Diaphanospondylodysostosis |
| *DCLRE1C* | c.1902_1903del | p.(Ser635Phefs*5) | Frameshift | rs760288938 | AR | Het | Severe combined immunodeficiency |
| *DTNA* | c.1757C>T | p.(Pro586Leu) | Missense | rs145425478 | AD | Het | Left ventricular noncompaction 1 |


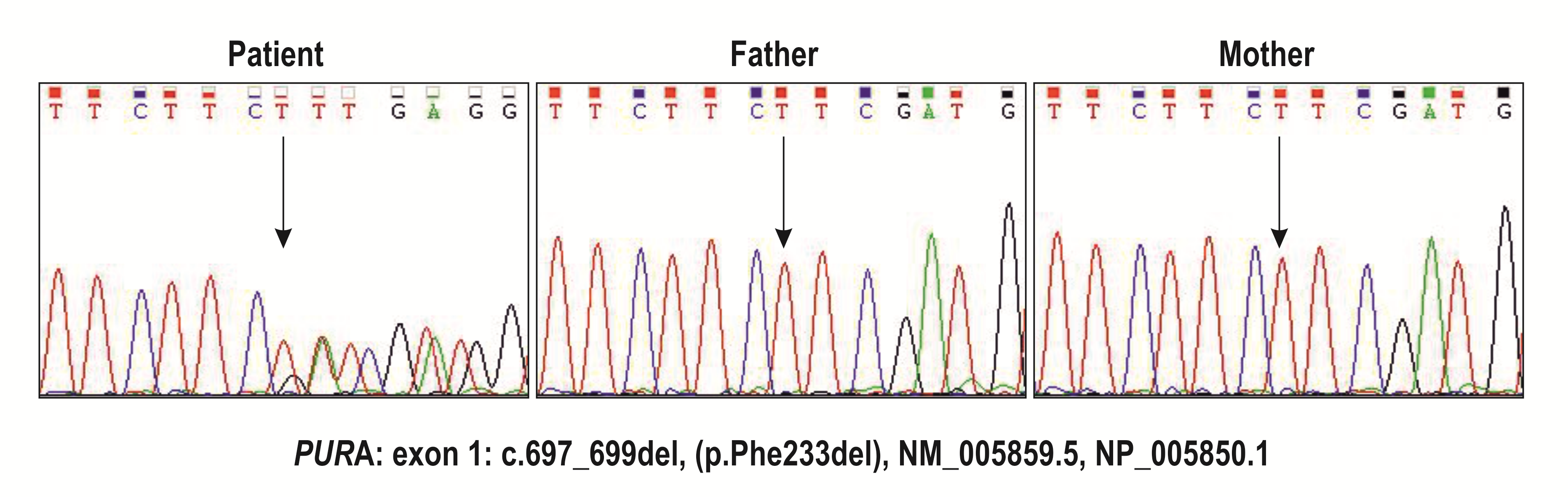
**Supplementary Figure 1**. **Molecular findings**. Sequence chromatograms showing the position of the de novo c.697_699del (p.Phe233del) variant (arrow) identified in heterozygosity in *PURA* (ref.seq. NM:005859.5, NP_005850.1).

| **Supplementary Table 3. Mutational spectrum involving *PURA*** | | | | | | | | | |
| --- | --- | --- | --- | --- | --- | --- | --- | --- | --- |
| **Patients** | **Sex** | **Age at evaluation** | **Chromosome locus** | | **Deletion size** | **Coordinates (hg19)** | | **Decipher** | **References** |
| 1 | M | 18m | 5q31.2-q31.3 | | 5,04 Mb | 137510879-142594536 | | 253734 | [Shimojima et al., 2011] |
| 2 | F | 8y | 5q31.2-q31.3 | | 2,57 Mb | 139137254-141702373 | | 4681 |
| 3 | M | 6y | 5q31.2-q31.3 | | 5,38 Mb | 138919021-144295587 | | - | [Hosoki et al., 2012] |
| 4 | F | 12y | 5q31.2-q31.3 | | 1,16 Mb | 139239400-140398993 | | - |
| 5 | M | 15m | 5q31.2-q31.3 | | 1,58 Mb | 137983535-139612636 | | 248784 |
| 6 | F | 6y | 5q31.2-q31.3 | | 3,2 Mb | 137041315-140450545 | | - | [Brown et al., 2013] |
| 7 | M | 2,5y | 5q31.2-q31.3 | | 1,9 Mb | 139442775-141329275 | | - |
| 8 | F | 26y | 5q31.2-q31.3 | | 360 kb | 139308862-139669265 | | - | [Bonaglia et al., 2015] |
| 9 | M | 2y | 5q31.2-q31.3 | | 2,2 Mb | 137851716-140085772 | | - | [Shimojima et al., 2018] |
| **Patients** | **Sex** | **Age at evaluation** | **cDNA** | **Protein** | | **Type** | **Domain** | **dbSNP** | **References** |
| 1 | M | 6m | c.812_814del | p.(Phe271del) | | Deletion | PUR III | rs587782991 | [Lalani et al., 2014] |
| 2 | M | 7m | c.307_308del | p.(Ser103Hisfs*97) | | Frameshift | PUR I | rs587782992 |
| 3 | M | 10m | c.556C>T | p.(Gln186*) | | Nonsense | PUR II | rs587782993 |
| 4 | F | 21m | c.289A>G | p.(Lys97Glu) | | Missense | PUR I | rs587782994 |
| 5 | F | 23m | c.299T>C | p.(Leu100Pro) | | Missense | PUR I | rs587782995 |
| 6 | F | 2y | c.363C>G | p.(Tyr121*) | | Nonsense | PURI | rs587782996 |
| 7 | F | 2y | c.783C>G | p.(Tyr261*) | | Nonsense | PUR III | rs587782997 |
| 8 | F | 5y | c.470T>A | p.(Met157Lys) | | Missense | PUR II | rs587782998 |
| 9 | M | 12y | c.265G>C | p.(Ala89Pro) | | Missense | PUR I | rs587782999 |
| 10 | F | 12y | c.263_265del | p.(Ile88_Ala89delinsThr) | | Deletion | PUR I | rs587783000 |
| 11 | F | 15y | c.596G>C | p.(Arg199Pro) | | Missense | PUR II | rs587783001 |
| 12 | F | 4y | c.726_727del | p.(Phe243Tyrfs*50) | | Frameshift | PUR III | rs786204833 | [Hunt et al., 2014] |
| 13 | F | 14y | c.847del | p.(Glu283Argfs*45) | | Frameshift | Glutamine/Glutamate-rich | - |
| 14 | F | 12y | c.616A>T | p.(Ile206Phe) | | Missense | PUR II | rs786204834 |
| 15 | F | 6y | c.697_699del | p.(Phe233del) | | Deletion | PUR III | rs786204835 |
| 16 | M | 8y | c.563T>C | p.(Ile188Thr) | | Missense | PUR II | rs793888527 | [Tanaka et al., 2015] |
| 17 | M | 4y | c.768dup | p.(Ile257Hisfs*37) | | Frameshift | PUR III | rs793888532 |
| 18 | F | 10y | c.1A>T | p.(Met1?) | | Start-loss | - | rs793888530 |
| 19 | F | 6m | c.697_699del | p.(Phe233del) | | Deletion | PUR III | rs786204835 |
| 20 | F | 15y | c.4_8del | p.(Ala2Profs*197) | | Frameshift | - | rs793888537 |
| 21 | F | 5y | c.302_310del | p.(Thr101_Ser103del) | | Deletion | PUR I | rs793888533 |
| 22 | M | 10y | c.331_342del | p.(Arg111_Leu114del) | | Deletion | PUR I | - | [Okamoto et al., 2017] |
| 23 | F | 2y | c.808_809del | p.(Thr270Lysfs*23) | | Frameshift | PUR III | rs886041600 | [Rezkalla et al., 2017] |
| **Supplementary Table 3. Mutational spectrum involving *PURA*** | | | | | | | | | |
| **Patients** | **Sex** | **Age at evaluation** | **cDNA** | **Protein** | | **Type** | **Domain** | **dbSNP** | **References** |
| 24 | F | 17y | c.734G>C | p.(Arg245Pro) | | Missense | PUR III | - | [Reijnders et al., 2018] |
| 25 | M | 24y | c.235C>T | p.(Gln79*) | | Nonsense | PUR I | - |
| 26 | F | 9y | c.220T>C | p.(Tyr74His) | | Missense | PUR I | - |
| 27 | M | 12y | c.697_699del | p.(Phe233del) | | Deletion | PUR III | rs786204835 |
| 28 | F | 18y | c.697_699del | p.(Phe233del) | | Deletion | PUR III | rs786204835 |
| 29 | M | 22y | c.675_676insA | p.(Val226Serfs*68) | | Frameshift | PUR III | - |
| 30 | M | 1y | c.25G>T | p.(Glu9*) | | Nonsense | Glycine-rich | - |
| 31 | F | 4y | c.802G>T | p.(Gly268*) | | Nonsense | PUR III | - |
| 32 | M | 6y | c.572C>T | p.(Pro191Leu) | | Missense | PUR II | rs1064795165 |
| 33 | F | 13y | c.677_678del | p.(Val226Glyfs*67) | | Frameshift | PUR III | rs1064796830 |
| 34 | M | 21m | c.338_341dup | p.(Gly115Profs*87) | | Frameshift | PUR I | - |
| 35 | F | 14y | c.746_749dup | p.(Lys250Asnfs*45) | | Frameshift | PUR III | - |
| 36 | F | 4y | c.158 _159del | p.(Gly53Alafs*147) | | Frameshift | Glycine-rich |  |
| 37 | M | 9y | c.697_699del | p.(Phe233del) | | Deletion | PUR III | rs786204835 |
| 38 | M | 3y | c.734G>C | p.(Arg245Pro) | | Missense | PUR III | - |
| 39 | M | 5y | c.351dup | p.(Ile118Hisfs*83) | | Frameshift | PUR I | - |
| 40 | M | 25y | c.771_776del | p.(Ile257_Val259delinsMet) | | Deletion | PUR III | - |
| 41 | M | 4y | c.340del | p.(Leu114Trpfs*111) | | Frameshift | PUR I | - |
| 42 | M | 10y | c.493_507dup | p.(Gly165_Arg169dup) | | Duplication | PUR II | - |
| 43 | F | 3y | c.289A>G | p.(Lys97Glu) | | Missense | PUR I | rs587782994 |
| 44 | M | 7y | c.299T>G | p.(Leu100Arg) | | Missense | PUR I | - |
| 45 | F | 6y | c.127_130del | p.(Ser43Alafs*34) | | Frameshift | Glycine-rich | - |
| 46 | F | 23y | c.382C>T | p.(Gln128*) | | Nonsense | - | - |
| 47 | F | 16y | c.153del | p.(Leu54Cysfs*24) | | Frameshift | Glycine-rich | - |
| 48 | F | 18m | c.616_618del | p.(Ile206del) | | Deletion | PUR II | rs1064795428 |
| 49 | F | 30m | c.478A>T | p.(Lys160*) | | Nonsense | PUR II | - |
| 50 | F | 15y | c.711dup | p.(Asn238Glnfs*56) | | Frameshift | PUR III | rs886042017 |
| 51 | F | 11m | c.812_814del | p.(Phe271del) | | Deletion | PUR III | rs587782991 |
| 52 | M | 9y | c.135_138dup | p.(Gly47Argfs*155) | | Frameshift | Glycine-rich | - |
| 53 | F | 9y | c.808_809del | p.(Thr270Lysfs*23) | | Frameshift | PUR III | rs886041600 |
| 54 | F | 16y | c.159del | p.(Leu54Cysfs*24) | | Frameshift | Glycine-rich | - |
| 55 | M | 8m | c.685A>T | p.(Lys229*) | | Nonsense | PUR III | rs1057524637 |
| 56 | F | 5y | c.596G>C | p.(Arg199Pro) | | Missense | PUR II | rs587783001 | [Lee et al., 2018] |
| 57 | M | 14y | c.796A>T | p.(Lys266*) | | Nonsense | PUR III | rs793888529 |
| 58 | M | 1y | c.593dup | p.(Arg199Profs*2) | | Frameshift | PUR II | - |
| **Supplementary Table 3. Mutational spectrum involving *PURA*** | | | | | | | | | |
| **Patients** | **Sex** | **Age at evaluation** | **cDNA** | **Protein** | | **Type** | **Domain** | **dbSNP** | **References** |
| 59 | M | 4y | c.697_699del | p.(Phe233del) | | Deletion | PUR III | rs786204835 | [Lee et al., 2018] |
| 60 | M | 12y | c.419G>C | p.Arg140Pro | | Missense | - | rs793888536 |
| 61 | F | 1y | c.502del | p.(Leu168Cysfs*57) | | Frameshift | PUR II | - |
| 62 | F | 3y | c.458G>C | p.(Arg153Pro) | | Missense | PUR II | rs886039610 |
| 63 | M | 13y | c.264del | p.(Ile88Metfs*137) | | Frameshift | PUR II | rs1064793761 |
| 64 | M | 7y | c.218T>C | p.(Phe73Ser) | | Missense | PUR I | rs793888535 |
| 65 | F | 15y | c.382C>T | p.(Gln128*) | | Nonsense | - | - |
| 66 | F | 2y | c.745del | p.(Val249*) | | Nonsense | PUR III | rs793888539 |
| 67 | M | 11m | c.759T>G | p.(Tyr253*) | | Nonsense | PUR III | - |
| 68 | M | 8y | c.160_183dup | p.(Leu54_Leu61dup) | | Duplication | Glycine-rich | - |
| 69 | F | 27y | c.7_11del | p.(Asp3Argfs*196) | | Frameshift | - | - |
| 70 | F | 4y | c.449_456del | p.(Arg150Profs*48) | | Frameshift | PUR II |  |
| 71 | F | 13m | c.697_699del | p.(Phe233del) | | Deletion | PUR III | rs786204835 |
| 72 | M | 1y | c.367C>T | p.(Gln123*) | | Nonsense | - | rs886042229 |
| 73 | M | 10y | c.159del | p.(Leu54Cysfs*24) | | Frameshift | Glycine-rich | - |
| 74 | F | 2y | c.586del | p.(Ile196Serfs*29) | | Frameshift | PUR II | - | [Mayorga et al., 2018] |
| 75 | M | 19y | c.563T>C | p.(Ile188Thr) | | Missense | PUR II | rs793888527 | [Qiao et al., 2019] |
| 76 | M | 1m | c.733C>T | p.(Arg245*) | | Nonsense | PUR III | - | [Trau and Pizoli, 2020] |
| 77 | M | 17y | c.72del | p.(Gly25Alafs*53) | | Frameshift | Glycine-rich | - | [Rodríguez-García et al., 2020] |
| 78 | F | 4y | c.3G>A | p.(Met1?) | | Start-loss | - | - | [Jezela‐Stanek et al., 2020] |
| 79 | F | 4y | c.697_699del | p.(Phe233del) | | Deletion | PUR III | rs786204835 | Present study |
